# A retrospective review of birth outcomes at the Mother and Child Health Hospital in Lao People’s Democratic Republic, 2004–2013

**DOI:** 10.1186/s12884-016-1168-5

**Published:** 2016-11-28

**Authors:** Sonja J. Olsen, Phommady Vetsaphong, Phouvanh Vonglokham, Sara Mirza, Viengphone Khanthamaly, Touy Chanthalangsy, Seth Chittanavanh, Bounkong Syhavong, Ann Moen, Joseph Bresee, Andrew Corwin, Anonh Xeuatvongsa

**Affiliations:** 1 Influenza Division, Centers for Disease Control and Prevention, 1600 Clifton Road, Atlanta, GA 30329 USA; 2 Mother and Child Health Hospital, Vientiane, Lao PDR; 3 Ministry of Health, Vientiane, Lao PDR; 4 Influenza Program, CDC, Vientiane, Lao PDR; 5 The QED Group, American Embassy, Vientiane, Lao PDR

**Keywords:** Pregnancy outcome, Low birth weight, Preterm birth, Small for gestational age, Lao PDR

## Abstract

**Background:**

The Lao People’s Democratic Republic (Lao PDR) is a lower-middle income country making steady progress improving maternal and child health outcomes. We sought to ascertain if there have been improvements in three specific birth outcomes (low birth weight, preterm birth and small for gestational age) over the last decade.

**Methods:**

We retrospectively reviewed birth records between 2004 and 2013 at the Mother and Child Health (MCH) hospital in Vientiane. We defined preterm birth as gestation <37 weeks and low birth weight as <2,500 g. We calculated small for gestational age (SGA). We describe birth outcomes over time and compare proportions using Chi square.

**Results:**

Between 2004 and 2013, the annual average number of newborns delivered each year was 4,322 and the frequency of low birth weight ranged from 9.5 to 12%, preterm births from 6.3 to 10%, and infants born SGA from 25 to 35%. There were no improvements in these frequencies over time. Women <18 years at delivery had a statistically significantly higher frequency of babies born with a low birth weight (15.3 vs. 10.8%, *p* < 0.02) or preterm (16.4 vs. 7.8%, *p* < 0.01) than those aged >18. There was no difference in the frequency of babies born SGA by age (26.8% in women <18 years vs. 29.7% in women >18 years, *p* = 0.30).

**Conclusions:**

At the largest maternal and child hospital in Lao PDR, we found a high frequency of poor birth outcomes with no improvements over the last decade.

## Background

Improving maternal and child health are priorities for the United Nation’s Millennium Development Goals (MDG), and the World Health Organization (WHO) recommends various measures, including antenatal care, delivery in health facilities with skilled attendants, malaria chemoprophylaxis and tetanus toxoid vaccine during pregnancy, and childhood vaccination to achieve targets in key health indicators. The Lao People’s Democratic Republic (Lao PDR) is a lower-middle income country in Southeast Asia that has made numerous investments in child and maternal health over the last decade that have yielded substantial gains in key health indicators [1]. Maternal mortality rates declined from 796 deaths per 100,000 live births in 1995 to 357 in 2009 [2]. Mortality among children <5 years declined from 170 per 1,000 live births in 1993 to 79 in 2011 [2]. Although infant mortality rates fell during this time period from 114 to 68 per 1,000 live births, rates still exceed the defined target of 45 [2]. Since 2002, with substantial investment by Gavi, a global Vaccine Alliance of public and private partners to improve access to vaccines in less wealthy countries, Lao PDR’s national immunization program has expanded coverage of childhood vaccines; vaccine coverage for DTP3 increased from 53% in 2002 to 87% in 2013 and influenza vaccine was introduced in pregnant women in 2012 [3–5].

While these improvements are significant and bring Lao PDR closer to reaching MDG targets, Lao PDR still has one of the highest <5 mortality in the region, and infant and maternal mortality rates remain among the highest in the region. Furthermore, there is no information on whether there have been improvements in birth outcomes such as the proportion of infants born low birth weight, preterm or small for gestational age. To ascertain if there were improvements in birth outcomes over time we reviewed historical data from the largest maternal hospital in Lao PDR between 2004 and 2013. Reviewing these data may help us better understand birth outcomes patterns in the context of a country going through substantial economic development striving to reach their MDG goals.

## Methods

### Study design and population

The Mother and Child Health (MCH) hospital is a 70-bed referral hospital located in Vientiane, Lao PDR. There are five large delivery hospitals in Vientiane and approximately one third of all hospital deliveries are in MCH [6]. We conducted a retrospective cross sectional review of hospital records of live births delivered at MCH hospital between 2004 and 2013. Routine hospital practice was to record all deliveries into a logbook with one line for each infant; each entry had age of the mother, gravida, date of delivery, sex of the baby, gestational age and birth weight of the infant. We abstracted data only on live births; stillbirths were excluded. Some log books were missing including all from the year 2007. For births occurring in years 2004–2010, we entered all variables into a database; however, upon analysis we recognized that date of birth accuracy was limited to year only. In order to look at monthly birth patterns we then entered birth data for years 2011–2013. During this time period, we recorded only singleton births and captured date of delivery, sex of the baby, gestational age and birth weight of the infant. Using data from the hospital statistical office, we also recorded the total number of newborns delivered at MCH hospital in 2004–2011.

### Data analysis

Data were entered into Excel (Microsoft Office 2010) and Access (Microsoft Office 2010) and imported into SPSS (IBM SPSS Statistics, version. 21) for analysis. In 196 (1.4%) newborn records, gestational age was recorded as a two-week range; we selected the earlier week. We defined preterm birth as gestation <37 weeks and low birth weight as <2,500 g. We calculated small for gestational age (SGA) and large for gestation age (LGA) using the Kramer method [7]. SGA was defined as a live birth with a birth weight less than the 10^th^ percentile of birth weights of the same sex and same gestational age in weeks, and is expressed as a percentage of live births with gestational ages from 22 to 43 weeks. LGA was defined as a live birth with a birth weight more than the 90^th^ percentile of birth weights of the same sex and the same gestational, expressed as a percentage of live births with gestational ages from 22 to 43 weeks. For each individual newborn record in 2004–2010, there was no indication on whether it was a singleton or part of a multiple birth so all births were assumed to be singletons for the calculation of SGA and LGA. We used three month moving averages to smooth the curves for the proportion of birth outcomes small for gestational age, low birth weight and preterm. To compare proportions we used Chi-square and for multiple samples we used the Mantel-Haenszel test of linear association; to compare continuous variables we used the Mann-Whitney test for two independent samples and the Kruskal-Wallis test for k independent samples. A *p* < 0.05 was considered significant.

## Results

**Fig. 1:**
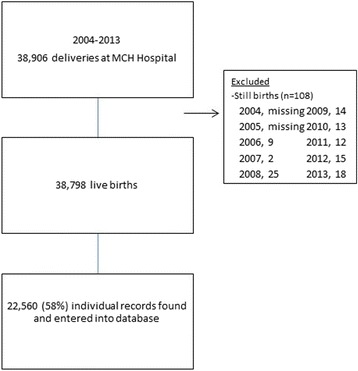
Flow diagram of deliveries at Mother and Child Health Hospital and live births included in retrospective review, Vientiane, Lao PDR

Between 2004 and 2013, the total number of recorded deliveries at MCH Hospital was 38,906 (Fig. 1). Excluding still births, there were 38,798 live births. Individual records on live births were available on 22,560, or 58% of all live births reported; these records were abstracted and entered into a database. The annual average number of live births at MCH Hospital was 4,311 and ranged from 3,951 in 2006 to 4,898 in 2012 (Table 1). The number of births in the dataset each year ranged from 874 in 2008 to 4,263 in 2011. In all live births for which we found data, there was >90% completeness for each variable (data not shown).

**Table 1:** Maternal characteristics and birth measures in the Maternal and Child Hospital, Lao PDR by year, 2004–2013

Year	Total no. live births at MCH hospital	No. (%) of live births in dataset	Mean age of mother (standard deviation)	Mean no. pregnancies (standard deviation)	Mean age in years at first pregnancy (standard deviation)	Mean birth weight in g (standard deviation)	% <2,500 g	Mean gestational age in weeks (standard deviation)	% <37 weeks	% SGA	% LGA
2004	missing	2027	26 (5.3)	2 (1.1)	23 (4.1)	3019 (486)	9.7%	39 (2.2)	6.3%	29%	2.4%
2005	4183	1087 (26)	26 (5.2)	2 (1.1)	23 (4.2)	2950 (483)	12%	39 (2.2)	9.8%	35%	4.6%
2006	3951	2075 (52)	25 (5.3)	2 (1.0)	24 (4.9)	3000 (466)	11%	39 (1.8)	6.0%	35%	3.5%
2007	4255	missing									
2008	4203	874 (21)	26 (5.6)	2 (1.0)	23 (4.4)	3005 (492)	11%	39 (2.2)	9.6%	27%	3.4%
2009	3994	2573 (64)	26 (5.2)	2 (1.1)	23 (4.0)	3007 (526)	12%	38 (2.1)	9.6%	25%	2.1%
2010	4317	1521 (35)	26 (5.5)	2 (1.6)	23 (4.7)	3015 (478)	11%	39 (1.9)	8.4%	31%	3.6%
2011	4277	4263 (99)	NA	NA	NA	3051 (455)	9.6%	39 (1.7)	7.1%	26%	2.6%
2012	4898	4228 (86)	NA	NA	NA	3029 (496)	10.7%	38 (2.0)	10%	25%	3.8%
2013	4720	3912 (83)	NA	NA	NA	3049 (454)	9.5%	39 (1.9)	8.1%	25%	3.5%

Maternal information was available in years 2004 through 2010 (Table 1). The annual mean age of mothers delivering was 26 years for all years except 2006, when it was 25 years. Of the 13,051 women with a known age, 275 (2.1%) were <18 years old. The annual mean number of lifetime pregnancies per woman was two, and the annual mean age at first pregnancy was 23 years for all years except 2006 when it was 24.

Data on infants were available for all years 2004–2013 (Table 1). The annual mean birth weight was 3,014 g with a statistically significant difference between years (*p* < 0.001), but birth weight was 40 g more in the years that only included singleton births (3043 g [SD 469] in 2011–2013 vs. 3003 g [SD 492] in 2004–2010, *p* < 0.001). The frequency of low birth weight (<2,500 g) ranged from 9.5% in 2013 to 12% in 2005 and 2009; there was no linear association with delivery year (*p* = 0.08). The annual mean gestational age was 38.8 weeks, and the frequency of preterm births ranged from 6.3% in 2004 to 10% in 2012; the linear association with delivery year was statistically significant (*p* = 0.002). The proportion of infants born SGA ranged from 25% in 2009, 2012 and 2013 to 35% in 2005 and 2006; the linear association with delivery year was statistically significant (*p* < 0.001).

**Fig. 2:**
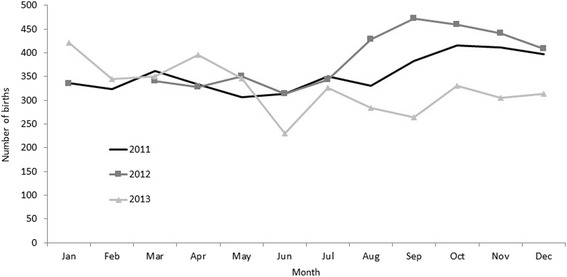
Number of live births by month and year at the Mother and Child Health Hospital, Vientiane, Lao PDR, 2011–2013

**Fig. 3:**
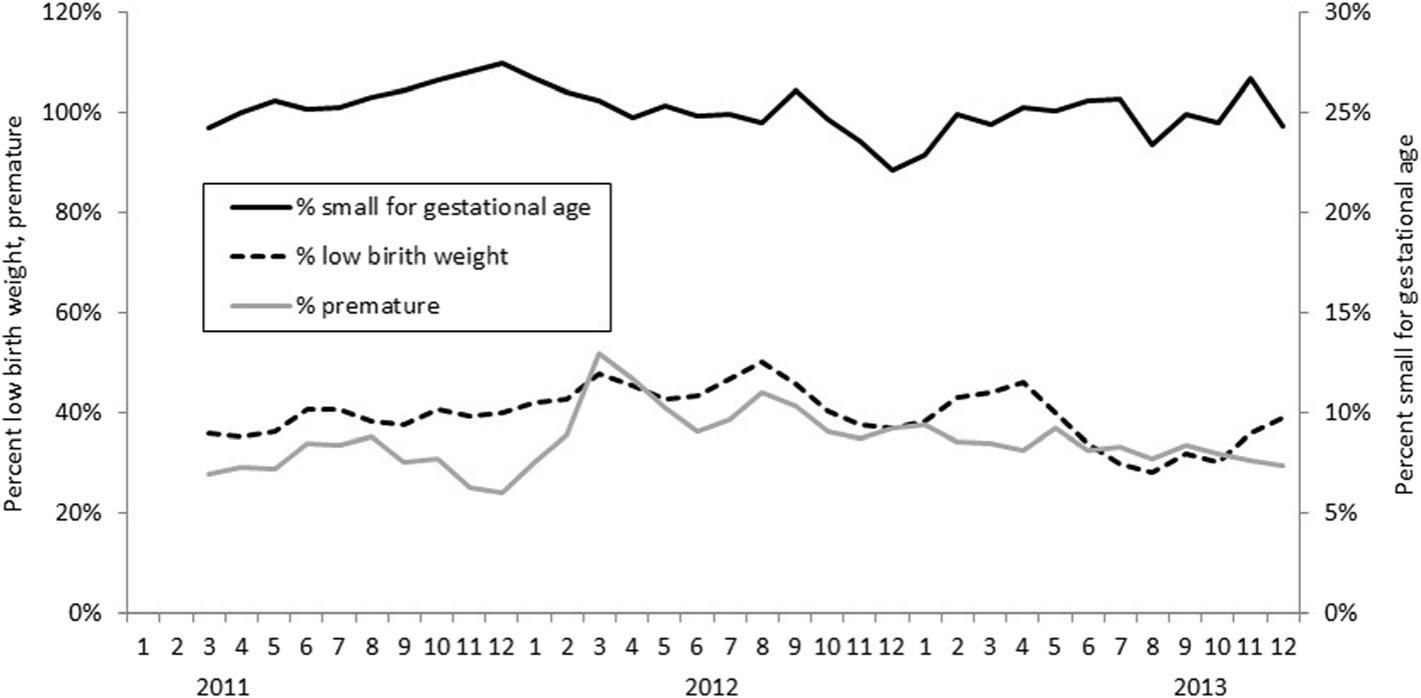
Live births at the Mother and Child Health Hospital that were a) percent small for gestational age, and b) by percent preterm and percent low birth weight Vientiane, Lao PDR, by month and year 2004–2011 (Note: data were missing for February 2012)

In years 2004 through 2010, for which we had maternal age at delivery, women <18 years old had a statistically significantly higher frequency of babies born with a low birth weight (15.3 vs. 10.8%, *p* < 0.02) or preterm (16.4 vs. 7.8%, *p* < 0.01) than those aged ≥18 old. There was no difference in the frequency of babies born small for gestational age by age (26.8% in women <18 years vs. 29.7% in women ≥18 years, *p* = 0.30). In years 2011–2013, we had monthly data on birth outcomes and observed an annual increase in the number of births in September through November in 2011 and 2012 but not in 2013 (Fig. 2). There was little variation in the monthly proportion of births born small for gestational age, preterm or low birth weight (Fig. 3).

## Discussion

At the largest maternal and child hospital in Lao PDR, we found a high frequency of poor birth outcomes with no improvement over the last decade and increases in the proportions of infants born preterm and SGA. In 2013, a quarter of infants were born small for gestational age, 9.5% low birth weight and 8.1% preterm.

Although many factors can affect birth outcomes, during this time there was substantial economic development in Lao PDR. Between 2004 and 2013, the gross national income (GNI) per capita increased from $390 to $1,490, and Lao PDR is now considered a lower-middle income country [1]. It remains just barely eligible for Gavi funds (GNI ≤ $1580). Despite these substantial economic improvements, we did not observe any improvement in birth outcomes over this time period in this population. There may be several explanations. First, urban migration has increased over time, bringing women in to Vientiane Capital from rural areas [8]. If general health status and prenatal care were worse in rural areas as indicated by a recent health survey [9] this population transition may temporarily keep some health indicators low in Vientiane. Alternatively, economic improvement may not have translated into better health outcomes. There have been some improvements in maternal and child health outcomes during this time period. For example, between 2004 and 2013, infant mortality decreased from 73 to 54 per 1,000 live births and under five morality decreased from 101 to 71 per 1,000 live births [1]. However, other health outcomes, such as the prevalence of stunting (44%) and underweight (27%) in children <5 years old remain exceptionally high [9]. Maternal nutrition is an important factor in birth outcomes, and the World Food Program (WFP) began in Laos in 2000. However, the targeted efforts of WFP in the very north and very south of the country may mean that the effects are not realized in the capital city [10].

Many factors, including maternal insults during pregnancy, contribute to poor birth outcomes such as infants being born small for gestational age. Infections, including influenza, in pregnant women are associated with higher risks of severe outcomes, [11] and maternal vaccination has been demonstrated to reduce the risk of infection in an infant born to a vaccinated mother during the first 6 months of life, an age at which the child is at very high risk of severe influenza disease [12–14]. Furthermore, recent studies have demonstrated that the vaccine may also play a role in birth outcomes, such as reducing the chance that a baby is born small for gestational age [11, 15]. In 2012, Lao PDR began targeting pregnant women, specifically at MCH hospital, for influenza vaccination [16].

These data have several limitations. First, data were abstracted from hard-copy logbooks, some of which had gone missing over the years. However, missing logbooks were unlikely to be drastically different from those we analyzed. Second, for all but the three most recent years of data, we lacked individual-level data on whether each newborn was a singleton or part of a multiple birth. Newborns that are part of multiple births are smaller and weigh less so inclusion in the calculation of SGA may have exaggerated the number of SGA infants. It may be that our population reference for calculating small for gestational age is not appropriate for Asian populations and overestimates the proportion of infants born small for gestational age. Nevertheless, with the consistent application of the methodology each year, any oberserved change was likely real. Finally, the hospital from which data were reviewed is the largest maternity hospital in Lao PDR located in Vientiane, a major urban city. It is likely that the women using this hospital may have better access to care than those from rural Lao PDR, and therefore the results may not be generalizable to rural Laos. Despite these limitations, the findings are consistent with data from other Southeast Asian nations, suggesting that our data may be fairly representative [17, 18].

In Lao PDR, prenatal health care, proper maternal health and nutrition and maternal education have all been demonstrated to be important factors to improve outcomes of infants [18]. Other factors, such as vaccinations, to prevent infections during pregnancy may also prove important. Our findings highlight the high burden of poor birth outcomes and the critical need for interventions.

## Conclusions

An analysis of 10-years of data on live births from the largest maternal and child hospital in Lao PDR found a consistent, high frequency of poor birth outcomes. Furthermore, we found increases in the proportions of infants born preterm and SGA. These results highlight the critical need for evidence-based interventions such as prenatal health care, proper maternal health and nutrition and maternal education.

## Abbreviations

GNI: gross national income
Lao PDR: Lao People’s Democratic Republic
LGA: Large for gestation age
MCH: Mother and Child Health
MDG: Millennium Development Goals
MoH: Ministry of Health
SGA: Small for gestational age
WFP: World Food Program
WHO: World Health Organization
